# Developing empathy in healthcare professions students: protocol of a mixed-methods non-controlled longitudinal intervention study

**DOI:** 10.3389/fmed.2024.1452516

**Published:** 2024-10-01

**Authors:** Andre Matthias Müller, Nicola Siew Pei Ngiam, Michael Dunn, Dujeepa D. Samarasekera, Benjamin Yen Seow Goh, Charlene En Hui Goh, Ann Toh, Jeannette Lee, Wai-Ping Yau, Lydia Siew Tang Lau, Paul John Gallagher

**Affiliations:** ^1^Saw Swee Hock School of Public Health, National University of Singapore, Singapore, Singapore; ^2^National University Hospital, Singapore, Singapore; ^3^Khoo Teck Puat National University Children’s Medical Institute (KTP-NUCMI), Singapore, Singapore; ^4^Centre for Biomedical Ethics, Yong Loo Lin School of Medicine, National University of Singapore, Singapore, Singapore; ^5^Centre for Medical Education, Yong Loo Lin School of Medicine, National University of Singapore, Singapore, Singapore; ^6^Department of Surgery, National University Hospital, Singapore, Singapore; ^7^Faculty of Dentistry, National University of Singapore, Singapore, Singapore; ^8^Yong Loo Lin School of Medicine, National University of Singapore, Singapore, Singapore; ^9^Department of Pharmacy and Pharmaceutical Sciences, Faculty of Science, National University of Singapore, Singapore, Singapore; ^10^Alice Lee Centre for Nursing Studies, Yong Loo Lin School of Medicine, National University of Singapore, Singapore, Singapore

**Keywords:** interdisciplinary instruction, healthcare education, psycho-social competencies, healthcare professionalism, compassion

## Abstract

Despite the necessary focus on clinical skills and knowledge during the tertiary education of healthcare professionals, the literature highlights the importance of developing psycho-social competencies. Empathy, a cognitive-behavioral attribute linked to various benefits for patients and healthcare professionals, is one such competency. Pedagogical approaches to successfully develop empathy in tertiary healthcare students are available. However, these approaches are often integrated piecemeal throughout the tertiary education journey. Research on a more empathy-focused curriculum is scarce. This manuscript describes the design of a study that aims to examine the effects of a more empathy-focused curriculum on empathy in tertiary healthcare profession students in Singapore. Freshmen dentistry, medicine, nursing, and pharmacy students enrolled in a novel curriculum with a strong empathy focus will be recruited for the study and followed for the program’s extent. Mixed-methods data collection at various time points will be conducted. Quantitative data will be collected on cognitive-behavioral empathy, intentions to provide empathic care, and engagement in courses of the curriculum. Qualitative data on perceptions of patient care and empathy in relation to relevant courses of the curriculum will be collected to provide context for quantitative findings. Ethics approval was granted by the Departmental Ethics Review Committee of the Saw Swee Hock School of Public Health, National University of Singapore (Ethics ID: SSHSPH-214).

## Introduction

In addition to clinical skills and knowledge, healthcare professionals need a range of psycho-social competencies to provide quality care (not merely cure) to patients. One of these professional competencies is empathy (1, 2). Although empathy has been widely studied in healthcare and healthcare education, there is considerable debate about its definition in the context of patient care (3, 4). Recently, however, the literature has converged toward a cognitive-behavioral view of empathy.

This view can be illustrated by Hojat who, after much research, defined empathy in the context of patientcare as “…a predominantly cognitive attribute that involves an understanding of experiences, concerns, and perspectives of the patient, combined with a capacity to communicate this understanding, and an intention to help” (3). This definition, along with others (2, 5, 6) emphasizes the cognitive aspect of empathy, which is argued to be the most relevant in patient-healthcare provider relationships. Cognition is emphasized because understanding requires advanced mental activities of perception, analysis and appraisal of information, and response generation (7). This process often involves recognizing as well as understanding of and responding to patients’ feelings, while regulating one’s own emotions to prevent burnout and maintain accuracy in judgment, among others (2, 8, 9).

Such higher-order cognitive processing allows health care professionals to gain a more accurate and non-judgemental understanding of patients leading to more appropriate responses. Consequently, supportive connections between healthcare professionals and patients are formed. These connections have various health- and well-being promoting effects driven by psycho- and socio-physiological processes occurring in supportive interpersonal relationships (3, 10–12). Empathic connections also translate into trust, leading to better cooperation between healthcare professionals and patients, facilitating early diagnosis, and treatment adherence as well as success (13–16). For healthcare professionals, meaningful empathic relationships can improve the sense of accomplishment and job satisfaction (8), while reducing stress and burnout (17–20), all of which are crucial for wellbeing. Given the high-stress working conditions of healthcare professionals and the associated negative outcomes such as exhaustion and burnout (21), the positive effects of empathic care are also essential in the context of protecting the healthcare system as a whole.

The myriad benefits of empathic engagement between healthcare professionals and patients highlight the need to develop and maintain empathy-related attitudes and competencies across all healthcare professional groups. Fortunately, empathy as we introduced it above, is a cognitive-behavioral attribute which can be developed through education (3, 6, 20, 22). Ideally, such education should begin early in training of healthcare professionals, with empathy-promoting elements to further nurture empathic care interspersed throughout the curriculum and beyond (23, 24). Since many healthcare profession students enroll in their courses to help others (3), it is crucial to capitalize on this teachable moment by incorporating empathy-promoting instruction early on. Further exposure to empathy education throughout training may increase competencies and prevent the drop in empathy observed in some healthcare professional students and in some contexts (25, 26). Reductions in empathy have been reported among medical students in the United States, the United Kingdom (27), New Zealand (28) and Iran (29), dental students in the United States (30) and Canada (31), nursing students in the United States (32) as well as medical, dental and nursing students in Trinidad and Tobago (33). In Singapore, where the study described in this manuscript is conducted, previous research revealed no significant changes in empathy among medical students throughout their 5 years of training (34).

Some evidence on effective pedagogical strategies to cultivate empathy in healthcare professionals and students is available. Generally, these strategies focus on understanding people, their circumstances, and needs, and developing interpersonal skills (3). Despite the importance of empathy and the availability of pedagogies to develop it, empathy is usually not explicitly targeted in the curricula for healthcare professional students (20, 27, 35). Instead, opportunities to develop socio-cognitive attitudes and skills related to empathy are offered throughout the education journey. This reality is reflected in the research literature as studies primarily report on the effects of short to medium-term interventions such as workshops or training modules (20, 22, 36). Research on a more empathy-focussed curriculum spanning several semesters at the beginning of tertiary healthcare training is currently scarce.

The research is also limited by the fact that the available scientific literature on empathy interventions in healthcare professions students is primarily from high-income countries in North America and Europe (35). Relevant research from Asia is notably scarce, with a recent review (20) identifying only one such study, conducted in China (37). A later review focusing on qualitative aspects of empathy education interventions (38) identified another study, also from China (39). We are aware of only one relevant intervention study conducted in Singapore; this work involved an evaluation of a short empathy-training course for pharmacy students (40).

Lastly, intervention studies are typically conducted in students from a single healthcare discipline (20, 22, 36, 38). For instance, Winter’s review (2020) identified only one study that included healthcare students from multiple disciplines (41). Relatedly, most studies focused on medical and nursing students, with less research on pharmacy students and very little on dental students (42).

We responded to the call for mixed-methods longitudinal intervention research on empathy in healthcare professions students (3, 6, 35) and conceived a study recruiting freshmen dentistry, medicine, nursing and pharmacy students at the National University of Singapore. The primary aim of our study is to examine the effects of consecutive courses of a novel Common Core Curriculum for healthcare professions students that emphasize understanding patients, communication, and other critical areas of professionalism on empathy. Our secondary aims are: (a) Examining the effects of the novel curriculum on intentions to provide empathic care, (b) Studying the association between engagement in courses of the novel Common Core Curriculum and empathy as well as intentions to provide empathy care, (c) Exploring perspectives on and experiences with courses of the new Common Curriculum in relation to empathy and patientcare, and (d) Examining trajectories of empathy and intentions to provide empathy care beyond the novel Common Core Curriculum.

## Materials and methods

The study involves multiple units within the National University of Singapore (NUS), including the Yong Loo Lin School of Medicine, the Alice Lee Centre for Nursing Studies, the Department of Pharmacy and Pharmaceutical Sciences and the Faculty of Dentistry.

We received ethics approval for this study from the Departmental Ethics Review Committee of the Saw Swee Hock School of Public Health, National University of Singapore (Ethics ID: SSHSPH-214).

### Design

This study is a longitudinal intervention study without a control group. We will use a mixed-methods approach, utilizing both quantitative and qualitative data to achieve our research objectives (43). Such an approach has been advocated for in healthcare education research (44). It is also warranted to account for the complexity of evaluating educational programs in general. Quantitative data will provide insights into the potential effects of the intervention on predefined outcomes (i.e., empathy), while qualitative research will provide context. Additionally, qualitative data can inform teaching practice of subsequent iterations of the Common Core Curriculum to improve the educational impact related to empathy.

**Figure 1:**
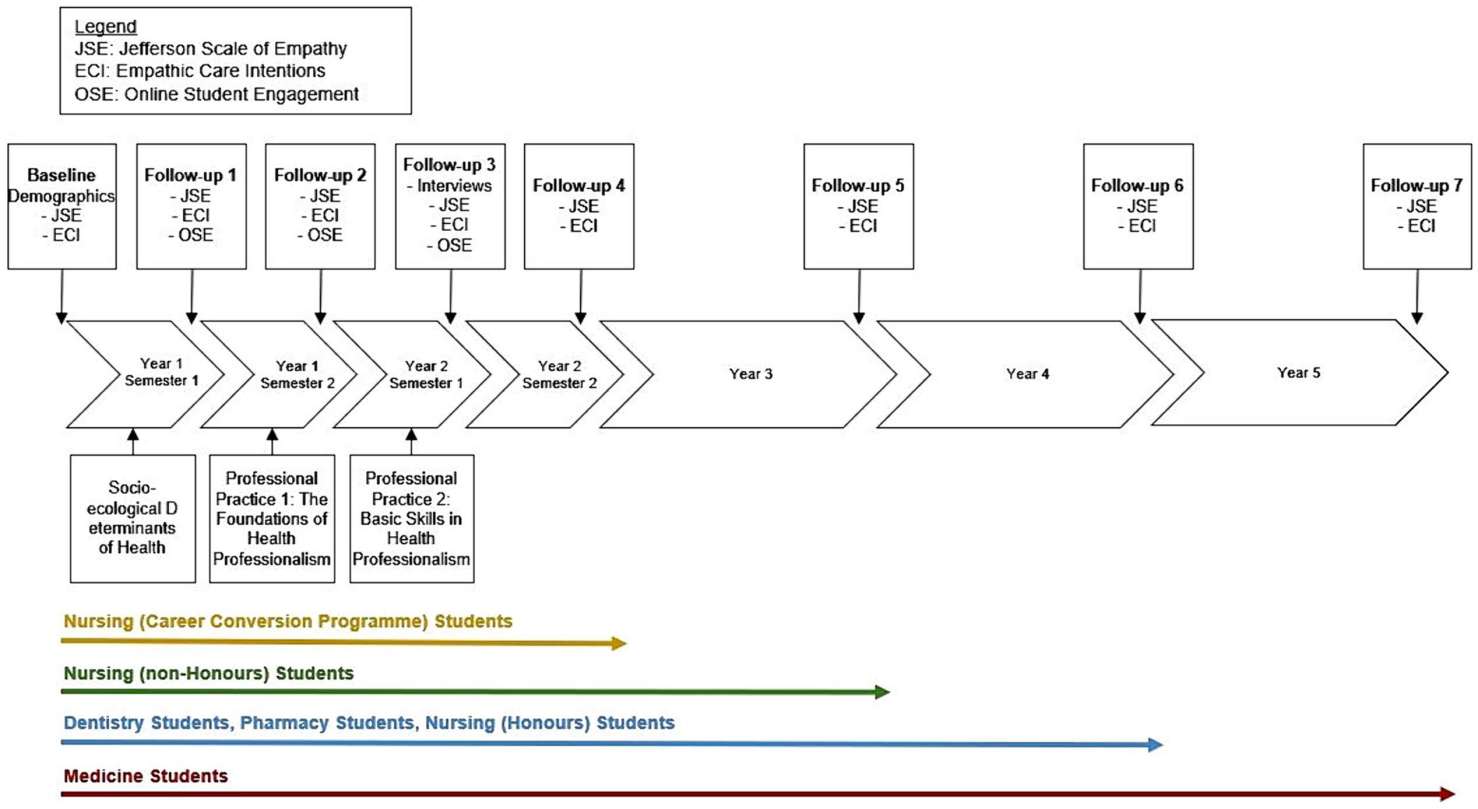
Overview of the overall study.

The study began in August of 2023 with the first wave of recruitment and will run for 6 years. Data collection time points are scheduled along the duration of the study programs: between 2 and 5 years depending on the program. Figure 1 provides an overview of the study, including information on study enrolment, delivery of intervention courses, and data collection time points as well as data collection methods.

### Participants

Participants in this study are freshmen dentistry, medicine, nursing, and pharmacy students at the National University of Singapore. There are no other inclusion criteria. All students enrolled in the respective programs will be invited to participate in this study (*n* = 900 per Academic Year). Participant Information Sheets are provided to all eligible students interested in joining the study. Students must provide consent before formally enrolling in each component of the research.

For the survey component, students will be enrolled in the study until their projected graduation. This means nursing, pharmacy and dentistry, and medical students will be enrolled for two to four, four, four and five years, respectively. For the interview component, a separate recruitment within the entire student cohort will be conducted, and consenting students will enroll for a single interview.

### Intervention

#### Overview

The intervention comprises three consecutive courses of the Common Core Curriculum for Health Professions Students which was fully implemented in August 2023. Course 1 is called Socio-ecological Determinants of Health; Course 2 is called Professional Practice 1: The Foundations of Health Professionalism; Course 3 is called Professional Practice 2: Basic Skills in Health Professionalism. All freshmen dentistry, medicine, nursing and pharmacy students enroll together in Course 1 in Semester 1 of their healthcare training; Courses 2 and 3 are conducted in subsequent semesters. Each course spans one semester of 13 weeks.

The three courses are explicitly and implicitly designed to enhance empathy-related attitudes and skills in students. Considering the cognitive-behavioral attributes and skills required for empathic engagement and the evidence on how these could be developed in healthcare professionals and students (3, 6, 26), important goals of the courses are to enhance students’ understanding of patients, professional identity formation, and interpersonal skills. The courses are sequenced to progressively deepen students understanding and skills related to empathy throughout their healthcare training. Course 1 focuses on understanding patients and their circumstances while Courses 2 and 3 are meant to develop interpersonal skills which are crucial to gain insights into patients and their lives (35). Various teaching and learning activities are designed to achieve the relevant goals in each course. In the next section we describe pedagogies implemented to develop empathy.

#### Pedagogies employed to develop empathy

##### Didactic training

Didactic training, commonly delivered in empathy development programs (26, 45) covers a range of topics that underscore the importance of empathy in healthcare professions. We introduce the socio-ecological view, communication strategies, ethical frameworks, and professional attributes. Collectively, these topics emphasize the need to understand and acknowledge the individuality of each patient, their circumstances, and perspectives to deliver effective care. Additional materials that reinforce the importance of empathy for healthcare professionals are also provided. Some evidence of efficacy of didactic training in this context is available (46).

##### Perspective taking exercises

To foster empathic understanding, we primarily use perspectives taking exercises. These exercises involve scenarios such as personal stories or recorded encounters with healthcare professionals. Students are encouraged to imagine the experiences and feelings of the people involved, effectively stepping into their shoes. Additional strategies include reflecting on students’ own experiences in healthcare settings, a concept known as the ‘wounded-healer effect’ (47). We also conduct role-playing activities to enhance perspective-taking capacity. For instance, students take on the roles of a healthcare professional who interview a patient struggling with a lifestyle behavior.

Taking the perspective of others not only builds capacity for understanding and empathizing with people who have diverse stories (48), but it also reduces the distance between patient and healthcare professional, laying the groundwork for empathic engagement. Studies involving healthcare professionals and students have provided evidence on the effects of perspective-taking exercises on empathic understanding and response (3, 49).

##### Communication and interpersonal skills training

Training in communication and interpersonal skills has frequently been linked to improvements in empathy among healthcare professions students (6, 20, 50). Our courses involve several aspects of communication training.

Understanding verbal and non-verbal expressions includes training students to listen, observe and recognize opportunities for empathic engagement. It also involves identifying the meanings of expressions such as change of gaze, silence and laughter. A common method for training is asking students to analyze patient-healthcare professional interactions.

Empathic communication tasks include activities where students are asked to communicate their understanding of the needs, concerns and feelings as expressed by others. This involves training of non-verbal and verbal expression that communicate understanding and empathy. Additionally, we explore the impact of one’s own personal biases and attitudes on the interpretation of a patient’s needs.

##### Exposure to arts and literature

We provide students with access to some forms of literature and arts such as fiction, poetry, drama and other works. The purpose of such exposure is for students to gain insights into human experiences, emotions, perceptions, values and cultures, and to understand the many ways in which feelings and experiences can be expressed (51). For example, students can read about patients’ complex emotional journeys through illness or the daily-life experiences of people with disabilities. Such stories are often less familiar to young students (52). Exposure to these materials, along with related reflective prompts, prepares students to listen to patients’ stories with curiosity, take their perspective, and engage empathically (52, 53).

##### Reflective exercises

We incorporate various activities that require students to contemplate their approach or viewpoint on caring for people. Reflective exercises encourage students to be cognisant of their experiences and potential biases. Engaging students in empathic reflection has been used in various empathy development programs and interventions (54), and has shown to be important to empathy development (20, 46).

#### Empathy strategies and the three courses of the Common Core Curriculum

##### Course 1: Socio-ecological Determinants of Health

This course focusses on the socio-ecological factors that influence a persons’ health and wellbeing (55). The aim is to provide students with a solid understanding of the various circumstances that relate to people’s health, and in doing so, develop empathy toward people in different situations. The premise of teaching the socio-ecological view is that knowledge of diverse factors impacting health, can motivate students to ask and listen carefully to fully understand the people they are serving. This understanding might also enable them to take the perspective of others. Students are also exposed to ways to support people based on their understanding of circumstances, feelings and experiences. Course 1 primarily focuses on the cognitive aspect of empathy, that is perspective taking. Supplementary Table S1 introduces teaching and learning activities related to the development of empathy incorporated into Course 1.

##### Courses 2 and 3: Professional Practice 1 and 2

These courses focus on equipping students with core learning related to professional practice: ethical analytic skills, legal knowledge, communication and team working skills, and the required components for individual professional identity formation. The courses operate as two, sequential parts to provide a common foundation for all health professional students. Further learning in professional practice is then spiraled upwards in the remaining years of students’ training, once they return to their uniprofessional educational contexts.

Developing empathy lies front and centre in these courses. Students are to develop an understanding of how empathy features as one core attribute of a healthcare professional’s identity. To develop such an understanding, fundamentals of therapeutic relationships with patients are explored through observation of recorded interactions with real patients, among others.

Furthermore, students are encouraged to act with empathy in the undertaking of specific learning activities; particularly regarding how they conduct themselves as learners in the classroom and within learning groups. In this sense, the premise of developing a basic understanding of professional practice is to cultivate certain attributes in how students behave in their learning roles. In this way, we expect students to develop the cognitive dimensions of empathy, taking the perspective of a future healthcare practitioner and understanding what empathy demands in that role.

Lastly, behavioral aspects of empathy are expected to develop through interpersonal communication training elements. This involves discussing biases that could inhibit empathic relationships and how to overcome these. Additionally, verbal and nonverbal communication techniques that enable empathic interactions are introduced and practiced. Supplementary Table S2 introduces teaching and learning activities related to the development of empathy incorporated into Course 2.

### Data collection—survey questionnaire

Survey questionnaire data will be collected at baseline and at various other time points: following the conclusions of Courses 1, 2 and 3, and annually until student graduation. Students will self-administer the instruments through the online platform, Qualtrics.

#### Demographic information

At baseline, we collect the following demographic information from study participants: course of study, gender (male, female, other), age in years, ethnicity (Chinese, Indian, Malay, Other), and housing type as a proxy for socio-economic status of the family (private property or large government housing unit, to medium size government housing unit, small-size government housing unit).

#### Empathy

The primary outcome of our study is cognitive empathy, as measured by the Jefferson Scale of Empathy, Healthcare Professions Student version (JSE-HPS). It appears to be the only available instrument designed specifically to measure empathy among healthcare professions students that is psychometrically sound (3). It is also the most commonly used instrument in this context (35). After conducting face and content validity assessments, the developers performed psychometric testing on a generic version intended for medical students. Using a sample of medical students, they established criterion validity, and the internal consistency was found to be desirable with a Cronbach’s alpha coefficient of 0.87 (56). Based on the generic version, three versions of the JSE were developed to allow administration in different populations: medical students, physicians and other healthcare professionals, and healthcare profession students. The psychometric properties of all versions were found to be reasonably comparable across locations (3). The JSE for medical students has been utilized in Singapore (34).

The JSE-HPS has been validated and used in students of various healthcare professions across geographical locations, including Asia (57–61). Students need to respond to 20 items using a 7-point Likert scale ranging from strongly disagree to strongly agree. To ensure the validity of responses, half of these items are negatively worded. The instrument measures three factors related to cognitive empathy: Perspective Taking-10 items, Compassionate Care-8 items, Standing in Patient’s Shoes-2 items. The JSE-HPS has been utilized in pharmacy students in Singapore (40).

To measure students’ intentions to provide empathic care, we drew from two existing instruments. First, the 12-item Reynolds Empathy Scale was developed in the United Kingdom for the nursing context (62). Scale development was based on patients’ perceptions of nurses’ behaviors that are helpful and unhelpful in showing empathy. Validity and reliability has been examined in terms of encounters with nurses (63, 64). Second, the 10-item CARE measure was developed to allow patients to assess their healthcare professionals’ empathic engagement (65, 66). The validity and internal consistency have been established in the United Kingdom (for medical doctors) by the developers of the instrument (66).

We developed our 7-item instrument as follows. First, we chose items that are directly related to key behavioral aspects of empathy as per the empathy definition introduced earlier (3). These are trying to understand patients (i.e., exploring feelings, giving space to communicate, listening; 3 items), communicating understanding (1 item) and acting upon understanding (i.e., ignoring feelings and views when providing care, support based on needs, focus only on data and facts; 3 items). Second, we used the stem ‘when I work as a healthcare professional, I will/want to’ to indicate future-directed self-assessment (i.e., intentions). Third, we adopted a 7-point Likert scale ranging from strongly disagree to strongly agree to align answering options with the JSE-HPS and allow for more granularity. An example item reads ‘When I work as a healthcare professional, I want to give patients space to tell their stories.’

#### Engagement in teaching and learning activities

Following each of the three courses, we administer an instrument to assess students learning engagement in various domains. The importance of engagement beyond the cognitive domain to achieve significant learning has been described (67, 68). For example, connecting with fellow learners can raise confidence and contribute to more positive attitudes toward a course. This, in turn, can raise the emotional desire to learn, resulting in more time and effort being invested in engaging with learning materials, ultimately impacting learning outcomes (69–71). Some suggestive evidence for the link between engagement and empathy has been reported in the context of medical students’ learning communities (72). Researchers observed that valuing aspects of social, collaborative and behavioral engagement was associated with empathy. As such, engagement in one or more domains may impact the development of empathy.

We drew 12 items from the Online Student Engagement Scale (OSE) and made minor modifications for our purpose (73). The instrument will enquire about the following domains of learner engagement: behavioral and cognitive engagement (learning efforts-5 items), emotional engagement (interest and commitment-4 items), social and collaborative engagement (contributing and connecting-3 items). Students will assess how well certain behaviors, thoughts and feelings describe them in relation to each course on a 5-point Likert scale ranging from ‘describes me perfectly’ to ‘does not describe me at all’. An example item reads ‘Participating actively in group-project discussions.’ Validity and internal consistency (alpha = 0.91) of the OSE are satisfactory as assessed in university students in the United States.

#### Free-text items

During data collection following each of the three courses under investigation, we include an open-ended question inviting students to share how the courses have impacted their views and skills around patientcare. Survey questionnaires for Follow-ups 4–7 include the following open-ended question: ‘What makes a good healthcare professional, please describe’. Free-text responses are meant to provide some context to the quantitative findings.

### Sample size and data analysis—survey questionnaires

#### Sample size calculation

We conducted sample size calculations to estimate the number of participants required for an adequately powered analysis of the effect of the three courses under investigation on empathy as measured with the JSE-HPS. Considering a small effect size of 0.2 (20), a power of 80% and a two-tailed significance level of 0.05 we would need 199 participants at Follow-up 3. To account for an expected significant drop-out of >50% over time we aim to recruit 400 students into the study.

#### Data analysis

To estimate the overall effect of the three courses on empathy and empathic care intentions across all students, we plan to conduct within-group t-tests. Repeated measures ANOVAs are planned to investigate the interactions between time and socio-demographic variables as well as engagement in teaching and learning activities on empathy and empathic care intentions. Significant interaction effects will be followed up with post-hoc tests and simple effects analysis as appropriate. Significant main effects will be followed up with post-hoc tests. The significance level will be set at 0.05. Data will also be plotted to visualize the results.

Free-text comments will be analyzed using qualitative thematic analysis (74). We will employ an inductive approach allowing the data to guide the generation of categories, themes and subthemes. We will first familiarize ourselves with the data by reading responses multiple times. Following this, we will conduct open coding of responses to capture all relevant content. We will then organize the codes into categories based on the overall topic they reflect. Within categories, we will identify themes and subthemes before crafting a narrative that will be supported by excerpts from the responses.

### Data collection—interviews

Interview procedures and reporting were guided by the Consolidated Criteria for Reporting Qualitative Research (COREQ) checklist (75).

Experienced qualitative researchers working in the healthcare or education sector will conduct semi-structured interviews following the completion of Course 3, marking the end of the Common Core Curriculum. Utilizing a pre-piloted interview guide, open-ended questions and prompts will be used to explore three broad areas with research participants: reasons for selecting their healthcare profession and experiences with the study program, perceptions and skills pertaining to patient care and empathy, and the contributions of the Common Curriculum courses on perceptions and skills related to patient care and empathy. Throughout the data collection period, the interview guide will be iteratively refined to explore emerging themes and topics previously not considered.

Interviews will be conducted in English via widely used videoconferencing platforms such as MS Teams or Zoom. Interviews will be audio-recorded using platforms built-in features. Field notes of interviews will supplement the audio recordings.

#### Sampling

We will employ a purposive sampling strategy to ensure diversity in experiences and perspectives are captured. We aim to recruit dentistry, medicine, nursing and pharmacy students until saturation is achieved within each student group. We project that 30 students in total will be sufficient. However, we are mindful that more participants may be needed to draw a rich picture.

#### Data analysis

Verbatim transcripts of interviews will be used for a primarily inductive thematic analysis (74). Briefly, following line-by-line coding of transcripts, we will develop and iteratively refine broad analytical categories, within which we will identify themes and subthemes. Representative quotes from participants will be selected to illustrate the findings. Depending on the data, we will highlight differences of themes and subthemes across students from different programs.

## Conclusion

Empathy as a cognitive-behavioral attribute is important across healthcare disciplines and should be developed throughout tertiary healthcare training. Pedagogical strategies to do this are widely available and most scientific studies point to desirable effects when such strategies are being examined, often in isolation (35). Researchers also suggest that empathy-related education is acceptable to healthcare professions students with many reporting related instruction to be valuable. Unfortunately, many curricula lack a distinct focus on developing the essential socio-cognitive competency of empathy, leaving much to be desired (35). This observation prompted a growing number of educators and education researchers to call for a meaningful integration of empathy training into the curriculum so as to nurture healthcare professionals who have the skills to cure, but also the attitudes and competencies to care (76–78).

With the mixed-methods longitudinal intervention study described in this manuscript, we will examine the effects of three consecutive courses of a novel Common Core Curriculum for Healthcare Professions Students on empathy in freshmen dentistry, medicine, nursing and pharmacy students enrolled at the National University of Singapore. Findings will provide insights into the potential of integrating empathy-related education into the curriculum for healthcare professions students and inform us about iterative changes we might need to make to enhance an anticipated impact.

## References

[1] Bellet PS. The importance of empathy as an interviewing skill in medicine. JAMA. (1991) 266:1831–2. doi: 10.1001/jama.1991.03470130111039, PMID: 1909761

[2] Larson EB. Clinical empathy as emotional labor in the patient-physician relationship. JAMA. (2005) 293:1100. doi: 10.1001/jama.293.9.1100 15741532

[3] Hojat M. Empathy in health professions education and patient care. Cham: springer International Publishing (2016).

[4] Scott H. Empathy in healthcare settings. Goldsmiths: University of London (2011).

[5] Berger DM. Clinical empathy. Northvale, New Jersey: Jason Aronson Inc (1987).

[6] Stepien KA Baernstein A. Educating for empathy. A review. J Gen Intern Med. (2006) 21:524–30. doi: 10.1111/j.1525-1497.2006.00443.x, PMID: 16704404 PMC1484804

[7] Schulte-Rüther M Markowitsch HJ Fink GR Piefke M. Mirror neuron and theory of mind mechanisms involved in face-to-face interactions: a functional magnetic resonance imaging approach to empathy. J Cogn Neurosci. (2007) 19:1354–72. doi: 10.1162/jocn.2007.19.8.1354, PMID: 17651008

[8] Gleichgerrcht E Decety J. Empathy in clinical practice: how individual dispositions, gender, and experience moderate empathic concern, burnout, and emotional distress in physicians. PLoS One. (2013) 8:e61526. doi: 10.1371/journal.pone.0061526, PMID: 23620760 PMC3631218

[9] Starcevic V Piontek CM. Empathic understanding revisited: conceptualization, controversies, and limitations. Am J Psychother. (1997) 51:317–28. doi: 10.1176/appi.psychotherapy.1997.51.3.317, PMID: 9327101

[10] Adler HM. The sociophysiology of caring in the doctor-patient relationship. J Gen Intern Med. (2002) 17:874–81. doi: 10.1046/j.1525-1497.2002.10640.x, PMID: 12406360 PMC1495122

[11] Christenfeld N Gerin W. Social support and cardiovascular reactivity. Biomed Pharmacother. (2000) 54:251–7. doi: 10.1016/S0753-3322(00)80067-0 10917462

[12] Uchino BN Cacioppo JT Kiecolt-Glaser JK. The relationship between social support and physiological processes: a review with emphasis on underlying mechanisms and implications for health. Psychol Bull. (1996) 119:488–531. doi: 10.1037/0033-2909.119.3.488, PMID: 8668748

[13] Hojat M Louis DZ Maio V Gonnella JS. Empathy and health care quality. Am J Med Qual. (2013) 28:6–7. doi: 10.1177/1062860612464731 23288854

[14] Nembhard IM David G Ezzeddine I Betts D Radin J. A systematic review of research on empathy in health care. Health Serv Res. (2023) 58:250–63. doi: 10.1111/1475-6773.14016, PMID: 35765156 PMC10012244

[15] Schneiderman LJ. Empathy and the literary imagination. Ann Intern Med. (2002) 137:627–9. doi: 10.7326/0003-4819-137-7-200210010-00033, PMID: 12353969

[16] Street RL Makoul G Arora NK Epstein RM. How does communication heal? Pathways linking clinician-patient communication to health outcomes. Patient Educ Couns. (2009) 74:295–301. doi: 10.1016/j.pec.2008.11.015, PMID: 19150199

[17] Chae SJ Jeong SM Chung Y-S. The mediating effect of calling on the relationship between medical school students' academic burnout and empathy. Korean J Med Educ. (2017) 29:165–73. doi: 10.3946/kjme.2017.62, PMID: 28870019 PMC5583431

[18] Lamothe M Boujut E Zenasni F Sultan S. To be or not to be empathic: the combined role of empathic concern and perspective taking in understanding burnout in general practice. BMC Fam Pract. (2014) 15:15. doi: 10.1186/1471-2296-15-15, PMID: 24456299 PMC3914722

[19] Shamasundar C. Understanding empathy and related phenomena. Am J Psychother. (1999) 53:232–45. doi: 10.1176/appi.psychotherapy.1999.53.2.232 10415993

[20] Winter R Issa E Roberts N Norman RI Howick J. Assessing the effect of empathy-enhancing interventions in health education and training: a systematic review of randomised controlled trials. BMJ Open. (2020) 10:e036471. doi: 10.1136/bmjopen-2019-036471, PMID: 32978187 PMC7520826

[21] Zuger A. Dissatisfaction with medical practice. N Engl J Med. (2004) 350:69–75. doi: 10.1056/NEJMsr031703 14702431

[22] Fragkos KC Crampton PES. The effectiveness of teaching clinical empathy to medical students: a systematic review and Meta-analysis of randomized controlled trials. Acad Med. (2020) 95:947–57. doi: 10.1097/ACM.0000000000003058, PMID: 31688037

[23] Dinoff A Lynch S Hameed AS Koestler J Ferrando SJ Klepacz L. When did the empathy die?: examining the correlation between length of medical training and level of empathy. Med Sci Educ. (2023) 33:489–97. doi: 10.1007/s40670-023-01768-1, PMID: 37251206 PMC10020755

[24] Ratka A. Empathy and the development of affective skills. Am J Pharm Educ. (2018) 82:7192. doi: 10.5688/ajpe7192, PMID: 30643318 PMC6325458

[25] Andersen FA Johansen A-SB Søndergaard J Andersen CM Assing HE. Revisiting the trajectory of medical students' empathy, and impact of gender, specialty preferences and nationality: a systematic review. BMC Med Educ. (2020) 20:52. doi: 10.1186/s12909-020-1964-5 32066430 PMC7027232

[26] Hojat M Axelrod D Spandorfer J Mangione S. Enhancing and sustaining empathy in medical students. Med Teach. (2013) 35:996–1001. doi: 10.3109/0142159X.2013.802300, PMID: 23758178

[27] Neumann M Edelhäuser F Tauschel D Fischer MR Wirtz M Woopen C . Empathy decline and its reasons: a systematic review of studies with medical students and residents. Acad Med. (2011) 86:996–1009. doi: 10.1097/ACM.0b013e318221e615, PMID: 21670661

[28] Lim BT Moriarty H Huthwaite M Gray L Pullon S Gallagher P. How well do medical students rate and communicate clinical empathy? Med Teach. (2013) 35:e946–51. doi: 10.3109/0142159X.2012.715783, PMID: 22938688

[29] Shariat SV Habibi M. Empathy in Iranian medical students: measurement model of the Jefferson scale of empathy. Med Teach. (2013) 35:e913–8. doi: 10.3109/0142159X.2012.714881, PMID: 22938682

[30] Sherman JJ Cramer A. Measurement of changes in empathy during dental school. J Dent Educ. (2005) 69:338–45. doi: 10.1002/j.0022-0337.2005.69.3.tb03920.x, PMID: 15749944

[31] Schwartz B Bohay R. Can patients help teach professionalism and empathy to dental students? Adding patient videos to a lecture course. J Dent Educ. (2012) 76:174–84. doi: 10.1002/j.0022-0337.2012.76.2.tb05244.x, PMID: 22319082

[32] Ward J Cody J Schaal M Hojat M. The empathy enigma: an empirical study of decline in empathy among undergraduate nursing students. J Prof Nurs. (2012) 28:34–40. doi: 10.1016/j.profnurs.2011.10.007 22261603

[33] Nunes P Williams S Sa B Stevenson K. A study of empathy decline in students from five health disciplines during their first year of training. Int J Med Educ. (2011) 2:12–7. doi: 10.5116/ijme.4d47.ddb0

[34] Samarasekera DD Lee SS Yeo SP Ponnamperuma G. Development of student empathy during medical education: changes and the influence of context and training. Korean J Med Educ. (2022) 34:17–26. doi: 10.3946/kjme.2022.216, PMID: 35255613 PMC8906926

[35] Byrne M Campos C Daly S Lok B Miles A. The current state of empathy, compassion and person-centred communication training in healthcare: an umbrella review. Patient Educ Couns. (2024) 119:108063. doi: 10.1016/j.pec.2023.108063, PMID: 38008647

[36] Kelm Z Womer J Walter JK Feudtner C. Interventions to cultivate physician empathy: a systematic review. BMC Med Educ. (2014) 14:219. doi: 10.1186/1472-6920-14-219, PMID: 25315848 PMC4201694

[37] Yang N Xiao H Cao Y Li S Yan H Wang Y. Does narrative medicine education improve nursing students' empathic abilities and academic achievement? A randomised controlled trial. J Int Med Res. (2018) 46:3306–17. doi: 10.1177/0300060518781476, PMID: 29976109 PMC6134671

[38] Winter R Leanage N Roberts N Norman RI Howick J. Experiences of empathy training in healthcare: a systematic review of qualitative studies. Patient Educ Couns. (2022) 105:3017–37. doi: 10.1016/j.pec.2022.06.015 35811257

[39] Potash JS Chen JY Lam CLK Chau VTW. Art-making in a family medicine clerkship: how does it affect medical student empathy? BMC Med Educ. (2014) 14:247. doi: 10.1186/s12909-014-0247-4 25431323 PMC4256925

[40] Han Z Barton KC Ho L-C Yap KZ Tan DS-Y Lee SS . Applying narrative medicine to prepare empathetic healthcare providers in undergraduate pharmacy education in Singapore: a mixed methods study. BMC Med Educ. (2024) 24. doi: 10.1186/s12909-024-05254-z PMC10943898 38491363

[41] Alhassan M. Effect of a 2-day communication skills training on nursing and midwifery students' empathy: a randomised controlled trial. BMJ Open. (2019) 9:e023666. doi: 10.1136/bmjopen-2018-023666, PMID: 30826757 PMC6429730

[42] Anishchuk S Kubacki A Howell Y van Harten MT Yarascavitch C MacGiolla PC. Can a virtual learning module foster empathy in dental undergraduate students? Eur J Dent Educ. (2023) 27:118–25. doi: 10.1111/eje.12783, PMID: 35114039 PMC10078759

[43] Creswell JW Plano CV. Designing and conducting mixed methods research. Thousand Oaks, CA: Sage Publications (2017).

[44] Schifferdecker KE Reed VA. Using mixed methods research in medical education: basic guidelines for researchers. Med Educ. (2009) 43:637–44. doi: 10.1111/j.1365-2923.2009.03386.x 19573186

[45] Lam TCM Kolomitro K Alamparambil FC. Empathy training: methods, evaluation practices, and validity. J Multidiscip Eval. (2011) 7:162–200. doi: 10.56645/jmde.v7i16.314

[46] Menezes P Guraya SY Guraya SS. A systematic review of educational interventions and their impact on empathy and compassion of undergraduate medical students. Front Med. (2021) 8:758377. doi: 10.3389/fmed.2021.758377, PMID: 34820397 PMC8606887

[47] Laskowski C Pellicore K. The wounded healer archetype: applications to palliative care practice. Am J Hosp Palliat Care. (2002) 19:403–7. doi: 10.1177/104990910201900611, PMID: 12442976

[48] Stephan WG Finlay K. The role of empathy in improving intergroup relations. J Social Isssues. (1999) 55:729–43. doi: 10.1111/0022-4537.00144

[49] Chen JT LaLopa J Dang DK. Impact of patient empathy modeling on pharmacy students caring for the underserved. Am J Pharm Educ. (2008) 72:40. doi: 10.5688/aj720240, PMID: 18483606 PMC2384215

[50] Kataoka H Iwase T Ogawa H Mahmood S Sato M DeSantis J . Can communication skills training improve empathy? A six-year longitudinal study of medical students in Japan. Med Teach. (2019) 41:195–200. doi: 10.1080/0142159X.2018.1460657, PMID: 29683011

[51] Oatley K. Scripts, transformations, and suggestiveness of emotions in Shakespeare and Chekhov. Rev Gen Psychol. (2004) 8:323–40. doi: 10.1037/1089-2680.8.4.323

[52] Hunter KM Charon R Coulehan JL. The study of literature in medical education. Acad Med. (1995) 70:787–94. doi: 10.1097/00001888-199509000-00016, PMID: 7669155

[53] Shapiro J Morrison E Boker J. Teaching empathy to first year medical students: evaluation of an elective literature and medicine course. Educ Health. (2004) 17:73–84. doi: 10.1080/13576280310001656196, PMID: 15203476

[54] Batt-Rawden SA Chisolm MS Anton B Flickinger TE. Teaching empathy to medical students: an updated, systematic review. Acad Med. (2013) 88:1171–7. doi: 10.1097/ACM.0b013e318299f3e3 23807099

[55] Marmot M Wilkinson R. Social determinants of health. 2nd ed. Oxford: Oxford University Press USA - OSO (2005).

[56] Hojat M Mangione S Nasca TJ Cohen MJM Gonnella JS Erdmann JB . The Jefferson scale of physician empathy: development and preliminary psychometric data. Educ Psychol Meas. (2001) 61:349–65. doi: 10.1177/00131640121971158

[57] Fields SK Mahan P Tillman P Harris J Maxwell K Hojat M. Measuring empathy in healthcare profession students using the Jefferson Scale of Physician Empathy: health provider--student version. J Interprof Care. (2011) 25:287–93. doi: 10.3109/13561820.2011.566648, PMID: 21554061

[58] Fjortoft N van Winkle LJ Hojat M. Measuring empathy in pharmacy students. Am J Pharm Educ. (2011) 75:109. doi: 10.5688/ajpe756109, PMID: 21931447 PMC3175671

[59] Hsiao C-Y Tsai Y-F Kao Y-C. Psychometric properties of a Chinese version of the Jefferson scale of empathy-health profession students. J Psychiatr Ment Health Nurs. (2013) 20:866–73. doi: 10.1111/jpm.12024 23205565

[60] Li L Wang J Hu X Hu X Xu C. Empathy in Chinese pharmacy undergraduates: implication for integrating humanities into professional pharmacy education. IJPER. (2015) 49:31–9. doi: 10.5530/ijper.49.1.5

[61] Williams B Brown T Boyle M Dousek S. Psychometric testing of the Jefferson scale of empathy health profession Students' version with Australian paramedic students. Nurs Health Sci. (2013) 15:45–50. doi: 10.1111/j.1442-2018.2012.00719.x, PMID: 23279312

[62] Reynolds WJ Scott B Jessiman WC. Empathy has not been measured in clients' terms or effectively taught: a review of the literature. J Adv Nurs. (1999) 30:1177–85. doi: 10.1046/j.1365-2648.1999.01191.x, PMID: 10564417

[63] Reynolds WJ. The measurement and development of empathy in nursing. Abingdon, Oxon: Routledge (2018).

[64] Yu J Kirk M. Evaluation of empathy measurement tools in nursing: systematic review. J Adv Nurs. (2009) 65:1790–806. doi: 10.1111/j.1365-2648.2009.05071.x, PMID: 19694842

[65] Mercer SW Maxwell M Heaney D Watt GC. The consultation and relational empathy (CARE) measure: development and preliminary validation and reliability of an empathy-based consultation process measure. Fam Pract. (2004) 21:699–705. doi: 10.1093/fampra/cmh621, PMID: 15528286

[66] Mercer SW McConnachie A Maxwell M Heaney D Watt GCM. Relevance and practical use of the consultation and relational empathy (CARE) measure in general practice. Fam Pract. (2005) 22:328–34. doi: 10.1093/fampra/cmh730, PMID: 15772120

[67] Pittaway SM Moss T. “Initially, we were just names on a computer screen”: designing engagement in online teacher education. AJTE. (2014) 39:140–156. doi: 10.14221/ajte.2014v39n7.10

[68] Redmond P Heffernan A Abawi L Brown A Henderson R. An online engagement framework for higher education. OLJ. (2018) 22:183–204. doi: 10.24059/olj.v22i1.1175

[69] Beachboard MR Beachboard JC Li W Adkison SR. Cohorts and relatedness: self-determination theory as an explanation of how learning communities affect educational outcomes. Res High Educ. (2011) 52:853–74. doi: 10.1007/s11162-011-9221-8

[70] Jones SM Kahn J. The evidence base for how we learn: Supporting Students' social, emotional, and academic development. Consensus Statements of Evidence from the Council of Distinguished Scientists. Aspen Institute. (2017). Available at: https://eric.ed.gov/?id=ed577039.

[71] Linnenbrink-Garcia L Pekrun R. Students’ emotions and academic engagement: introduction to the special issue. Contemp Educ Psychol. (2011) 36:1–3. doi: 10.1016/j.cedpsych.2010.11.004

[72] Tackett S Wright S Colbert-Getz J Shochet R. Associations between learning community engagement and burnout, quality of life, and empathy among medical students. Int. J. Med Educ. (2018) 9:316–22. doi: 10.5116/ijme.5bef.e834, PMID: 30504524 PMC6387776

[73] Dixson MD. Measuring student engagement in the online course: the online student engagement scale (OSE). OLJ. (2015) 19. doi: 10.24059/olj.v19i4.561

[74] Braun V Clarke V. Using thematic analysis in psychology. Qual Res Psychol. (2006) 3:77–101. doi: 10.1191/1478088706qp063oa

[75] Tong A Sainsbury P Craig J. Consolidated criteria for reporting qualitative research (COREQ): a 32-item checklist for interviews and focus groups. Int J Qual Health Care. (2007) 19:349–57. doi: 10.1093/intqhc/mzm042 17872937

[76] Engbers RA. Students' perceptions of interventions designed to foster empathy: an integrative review. Nurse Educ Today. (2020) 86:104325. doi: 10.1016/j.nedt.2019.104325, PMID: 31926381

[77] Bansal A Greenley S Mitchell C Park S Shearn K Reeve J. Optimising planned medical education strategies to develop learners' person-centredness: a realist review. Med Educ. (2022) 56:489–503. doi: 10.1111/medu.14707, PMID: 34842290 PMC9306905

[78] Bos-van den Hoek DW Visser LNC Brown RF Smets EMA Henselmans I. Communication skills training for healthcare professionals in oncology over the past decade: a systematic review of reviews. Curr Opin Support Palliat Care. (2019) 13:33–45. doi: 10.1097/SPC.0000000000000409, PMID: 30562180

