# Supplementary material for: Developing empathy in healthcare professions students: protocol of a mixed-methods non-controlled longitudinal intervention study
Source: Front Med (Lausanne). 2024 Oct 1;11:1452516. doi: 10.3389/fmed.2024.1452516 (PMC11473357; doi:10.3389/fmed.2024.1452516)
Supplement: Supplementary file 1 [file Table_1.DOCX]

**Table S1:** Teaching and learning activities meant to develop empathy as incorporated into Course 1 (Socio-ecological Determinants of Health)

| **Week** | **Teaching and Learning Activities** |
| --- | --- |
| Introduction, health and what impacts it | |
| 1 | *Perspective taking Exercise.*  Taking perspective of patient based on story of encounter with healthcare professionals. |
| 1 | *Didactic Training coupled with Perspective Taking Exercise.*  Learning about the biomedical and person-centred view of health, and imagining patients and healthcare professionals’ experiences and feelings when taking on each view |
| 1 | *Reflective Exercise.*  *Self-reflection on approach to providing care as future healthcare professional.* |
| *Block 1: The Socio-Ecological View and Health* | |
| 2 | *Didactic Training.*  Introduction to socio-ecological determinants and their impact on health. Highlighting how different circumstances relate to different health and wellbeing outcomes. |
| 2 | *Perspective Taking Exercise.*  Walk in the shoes of a 55-year person with low formal education and a cleaning job. Reflect about how this profile might impact various areas of health, and why so. |
| 2 | *Perspective Taking Exercise.*  Imagine you are part of a lower-income household and think about what it might take to improve health. |
| 3 | *Didactic Training coupled with Reflective Exercise*  Exploring the processes of how socio-ecological determinants impact health. Reflection on own family/social network impact health. |
| 3 | *Perspective Taking Exercise.*  Imagine you are 40 years old, have high formal education and work in a bank. How does your housing, social network, literacy look like, and how do these impact your health? |
| 3 | *Reflective Exercise.*  Students to imagine they are a healthcare professional. They need to reflect about how they can find out about a person’s circumstances that may impact certain health outcomes. |
| 4 | *Didactic training*  Highlighting how it is important to understand people to gauge health literacy and needs for social prescribing*.* |
| 4 | *Perspective Taking Exercise (Wounded-healer).*  Students reflect about interactions with healthcare professionals that left them confused. They also need to explore why they felt confused. |
| 4 | *Perspective Taking Exercise.*  Provision of stories of three people from different backgrounds. Students are asked to walk in their shoes and think about potential social prescriptions that could be helpful. |
| 5 | *Didactic training plus Perspective Taking Exercise*  Introduce the concept of marginalised populations and highlight the multiple intersecting barriers related to health.  Students need to imagine they are 22 years old and have spent the last 4 years in prison. They need to envision ecological barriers to health they encounter. |
| 5 | *Perspective Taking Exercise and Reflective Exercise*  Exposure to three stories of people who are marginalized. Students need to understand one person by mapping their socio-ecological determinants and explain how they impact health (for this they are encouraged to walk in the persons shoes). From that vantage point of understanding, they need to think of potential support option. A reflective prompt asks students to think about their implicit biases and how they could provide empathic care in the light of these. |
| Block 2: The Socio-Ecological View and Health Behaviors | |
| 6 | *Perspective Taking Exercise.*  Students are asked to conduct a role play. Students playing healthcare professionals need to walk in patients’ shoes when exploring the difficulties of engaging in lifestyle behaviors. They are to explore various factors to build understanding. A debrief where observers and patients feedback on communication approaches is conducted. |
| 7 | *Perspective Taking Exercise.*  Students chose a person in their family and assess screening behaviors. Based on the assessment, they need to walk in the persons shoes to understand reasons behind lack of screening. Person-centred support will need to be provided. |
| 8 | *Didactic Training.*  Introduction of a behavior change support model that acknowledge the various ecological barriers to health behavior adoption. |
| 8 | *Perspective Taking Exercise.*  Project groups watch an interaction between a healthcare professional and a patient. They are asked to imagine how the patient experiences the interaction and what the patient feels. |
| 9 | *Perspective Taking Exercise.*  Discussion prompt asks students to share how the course has impacted their views around what it means to be a healthcare professional. Students should discuss how different backgrounds and exposures impact views. This might allow students to see things from the perspective of other students. |
| Block 3: The Socio-Ecological View and Care Access, and upstream forces | |
| 10 | *Perspective Taking Exercise.*  Introduction of an elderly person who has various illnesses. Circumstances of this person are also provided. Students need to walk in the shoes of this person and imagine how this person might feel. Similarly, they are to imagine and articulate how health and healthcare access might be impacted by personal circumstances. |
| 11 | *Didactic Training (introduction to Longitudinal Patient Experience).*  Introducing an immersive learning element that bridges perspective taking in theoretical scenarios and perspective taking in the real world. |
| 11 | *Perspective Taking Exercise.*  Introduction of the narrative medicine approach, accompanied by exposure to an emotional poem by a migrant worker. Students are asked to put themselves into the shoes of the author and complete writing prompts (e.g., I wish…). |
| 12 | *No specific empathy-related elements provided.* |
| The Real World, Reflection and Summary | |
| 13 | *Perspective Taking Exercise*  Introduction to a real person from the community. Teaching staff shares a video of this person that also includes an interview and a community walk allowing the exploration of circumstances, realities, needs and support. Following the video, students examine socio-ecological factors impacting health and well-being of this person. Lastly, students will be prompted to walk in the person’s shoes and propose support options. |

**Table S2:** Teaching and learning activities meant to develop empathy as incorporated into Course 2 (Professional Practice 1: The Foundations of Health Professionalism)

| **Week** | **Teaching and Learning Activities** |
| --- | --- |
| Block 1: The Foundations of Health Professionalism | |
| 1 | *Didactic Training*  Learning about the specific work activities and responsibilities that different health professionals undertake. |
| 1 | *Perspective-taking Exercise.*  Team-based activity requiring each student to put themselves in the shoes of one of their team members, imagining what it would be like to train in a different profession. Self- and team-based reflections on what each different student from the different professions brings to the table, and how they envisage working together in training and in future practice. |
| 1 | *Reflective Exercise.*  Students to examine each professional attribute and to reflect on how the requirements of humility, integrity, respect, compassion and empathy will shape their involvement in the course, and how they will develop each attribute in working as a team engaging in the learning process. |
| 2 | *Didactic Training.*  Learning about an ethical framework for healthcare practice, consideration of how thinking ethically, and making ethical rather than simply technical judgments, invokes professional attributes and sensitivity to the recipient of care and the context of their lives. |
| 2 | *Perspective-taking Exercise.*  Case-based analysis requiring students to put themselves in the shoes of a junior health professional to think through two scenarios involving the prescription of opioids for a substance-dependent patient requiring surgery, and the provision of dental implants to a patient requesting them. |
| 3 | *Didactic Training*.  Core knowledge about how fitness to practice is conceptualised in professional regulation, and how professional standards are sensitised to both professional and personal standards of behaviour |
| 4 | *Didactic Training.*  Principles and elements of interpersonal and interprofessional relationships, and core competencies of interprofessional collaborative practice. |
| 4 | *Perspective-taking exercise.*  Students watch a recorded interaction of interprofessional healthcare team members discharging a patient from hospital and critique the elements of collaborative practice. This is followed by a case-based analysis of interpersonal communication techniques that impact a healthcare provider-patient relationship. |
| Block 2: The Basic Tenets of Effective Communication | |
| 5 | *Didactic Training.*  Learning about personal biases and values and how these may influence healthcare provider communication. Appreciating the importance of self-awareness in identifying personal biases and learning strategies to overcome these. |
| 5 | *Reflective exercise.*  Students are introduced to a model of reflective writing to help guide their reflection in a systematic and organised manner. They reflect on how personal biases may have impacted interactions with a patient either in their own experience or from what they have observed in the healthcare environment. |
| 6 | *Didactic Training*.  Learning about how to identify the barriers to effective communication and recognising the features of effective communication, and how to avoid communication breakdowns. |
| 6 | *Perspective-taking exercise.*  Group work to critique and role-paly improvements to a recorded interaction of a healthcare team, comprising of a doctor, dentist, nurse, and pharmacist, attending to a patient with an asthma attack at a dental appointment. |
| 7 | *Didactic Training*  Learning to recognise the elements of patient-centred communication and the importance of accurate history taking and a therapeutic alliance between healthcare provider and patient. |
| 7 | *Perspective-taking exercise.*  Video-based exploration of a patient’s narrative and the elements of effective therapeutic communication. Discussion around helpful and unhelpful behaviours in relationship-building, how to encourage a patient to tell her story, how to identify emotions and express appropriate empathy and the impact of the patient’s story on the student's thoughts and emotions. |
| 8 | *Didactic Training*  Review of the key principles and techniques of effective healthcare provider communication with patients and colleagues. |
| 8 | *Perspective-taking exercise.*  Hands-on interaction with a simulated patient and family member, focusing on demonstration of effective questioning techniques, active listening, non-verbal communication, and appropriate expression of empathy. |
| Block 3: Foundational Topics in Healthcare Ethics, Law and Professionalism | |
| 9 | *Reflective Exercise.*  Engagement with a personal narrative from a healthcare practitioner about the challenges faced around obtaining consent, engaging with family members, and communication strategies; students reflect on how they would do things differently in the face of encountering these challenges. |
| 9 | *Perspective Taking Exercise.*  Case-based analysis requiring one group to analyse a case involving a woman with breast cancer who is refusing a mastectomy. Students step into the shoes of the professional team managing this case to devise a legally, ethically and professionally appropriate course of action, and defend these decisions respectfully when challenges by other student groups in class. |
| 10 | *Didactic Training.*  Learning about circumstances in which it is justified to breach confidentiality, and the demands of a sensitive, respectful and empathetic approach to disclosing the decision to breach confidentiality to a patient. |
| 10 | *Reflective Exercise.*  A class debate about the appropriate professional management of confidential healthcare information for the purposes of student learning and training. |
| 11 | *Perspective Taking Exercise.*  Case-based analysis requiring two groups to analyse two cases a request for dental treatment after hours, and the prescription of steroids for a student athlete. Students step into the shoes of the professional team managing these cases to devise a legally, ethically and professionally appropriate course of action in relation to the core principle of acting in the patient’s best interests, and defend these decisions respectfully when challenges by other student groups in class. |
| 12 | *Didactic Training*.  Core knowledge about person-centred care and how finding ways to enable patients to share their personal narratives can enable patient-centredness and shared decision-making. |
| 12 | *Perspective Taking Exercise.*  Provision of a personal narrative by a patient with a chronic illness describing his experiences of receiving care and treatment from different professionals, and also 2 narratives provided by the patient’s health care team members. Students are required to identify the different perspectives that each person brings to understanding the patient’s circumstances and needs, and how these can differ depending on the different epistemic standpoints involved. |
| 12 | *Reflective Exercise.*  Small-group discussion focused on elucidating personal and team-based strategies for becoming person-centred in their approach to learning about patient care. |
| 13 | *No specific empathy-related elements provided.* |
